# Thymic Rosai-Dorfmann disease: a case report

**DOI:** 10.1186/s13019-024-02919-0

**Published:** 2024-06-21

**Authors:** Gaohua Liu, Min Jing, Juan Wang

**Affiliations:** 1 Department of Cardiothoracic Surgery, The First People’s Hospital of Neijiang, Sichuan, 641000 China; 2 Department of Pathology, The First People’s Hospital of Neijiang, Sichuan, 641000 China; 3 Department of Ultrasonic Medicine, The First People’s Hospital of Neijiang, No. 1866, West Section of Hanan Avenue, Shizhong District, Neijiang, Sichuan, 641000 China

**Keywords:** Thymus, Rosai–Dorfman disease (RDD), Surgery, Diagnose

## Abstract

**Background:**

Rosai-Dorfman disease (RDD), known as sinus histiocytosis with massive lymphadenopathy, commonly involves lymph nodes in the neck or mediastinum, although extranodal involvement is observed in approximately 40% of RDD patients. RDD involving only the thymus has rarely been reported. Here, we report a case of RDD originating in the thymus. The lesion was surgically removed, and a cure was finally achieved. There was no recurrence after telephone follow-up for 3 years.

**Case presentation:**

A 52-year-old male was accidentally found to have a 7 × 6 cm anterior mediastinum lump by chest computed tomography (CT). The mediastinal lesion was resected by surgery, and postoperative pathology revealed RDD originating from the thymus. Regular telephone follow-up after surgery lasted 3 years and showed that the patient remained in good condition without any relevant symptoms.

**Conclusions:**

RDD originating in the thymus cannot be characterized from CT images and is easily misdiagnosed as a traditional mediastinal tumor. This is mainly because there is so little disease in this area that physicians are not aware of it. We report this case with the hope that clinicians will have a better understanding of this disease. According to our follow-up results, surgery is an effective means of treatment.

## Case presentation

**Fig. 1:**
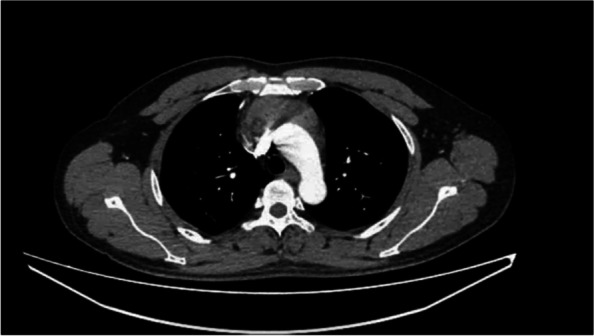
Contrast-enhanced CT scan showing partial enhancement in soft tissue, measuring 7 cm × 6 cm in size, in the anterior mediastinum

A 52-year-old man with a health examination revealing mediastinal space occupation was admitted to the hospital. The patient did not complain of fever, cough, flushing, palpitations, dyspnea, hoarseness, or eyelid drooping. No specific positive signs were found. A contrast-enhanced CT scan of the chest revealed a 7 × 6 cm anterior mediastinum lump adjacent to the mediastinal vessels (Fig. 1). No abnormities were found by hematologic examination, electrocardiography, or ultrasonic cardiography.

**Fig. 2:**
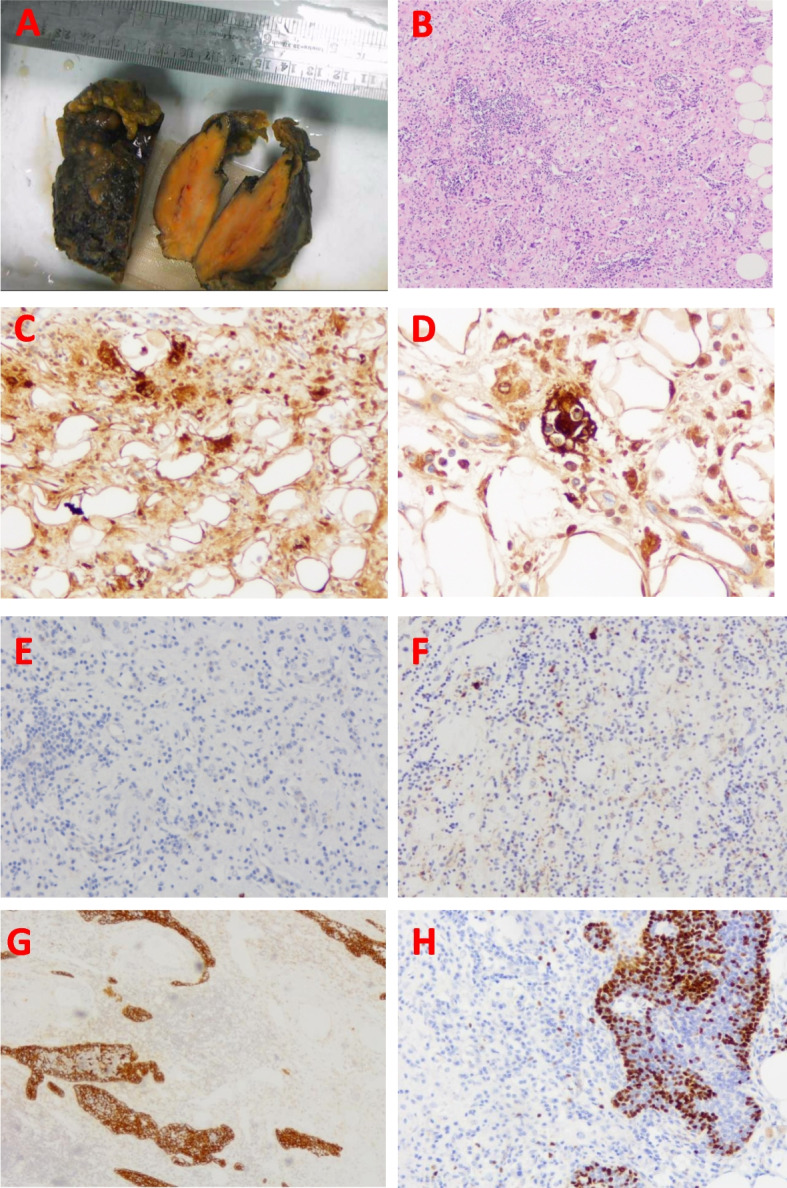
**A** The section of the tumor tissue was grayish yellow, solid and somewhat soft. **B** The lesion showed a "light and dark" area composed of many tissue cells, plasma cells and lymphocytes (HE, orginal magnification × 100). **C** Immunohistochemical staining for CD68 is positive. "Light area" shows large, cytoplasmic tissue cells (original magnification × 200). **D** Immunohistochemical staining for S-100 is positive. "Light area" showing scattered lymphocytes in cytoplasm of tissue cells, characteristic deep movement (original magnification × 400). **E** Immunohistochemical staining for CD1α is negative (original magnification × 200). **F** Immunohistochemical staining for CD 21 is positive negative (orginal magnification × 200). **G** Immunohistochemical staining for PCK is positive. Demonstrates residual thymic epithelium (original magnification × 100) **H** Immunohistochemical staining for TDT was positive, showing that lymphocytes were present in the remnant thymus tissue (original magnification × 100)

Hematological, electrocardiogram, and ultrasonic cardiogram tests were used to rule out surgical contraindications. Minimally invasive video-assisted thoracoscopy (VATS) was performed to resect the anterior mediastinal tumor. For safety reasons, VATS was intraoperatively converted to a traditional procedure through the use of a sternotome. Intraoperative exploration revealed tumor involvement of the right phrenic nerve and other adjacent mediastinal pleura and an incomplete and thickened envelope membrane around the lump. The tumor was completely removed. The patient developed right diaphragmatic paralysis and hypoxemia after surgery and was discharged from the hospital on the 7th postoperative day after active treatment with a noninvasive ventilator. Pathological examination revealed that the lesion was RDD. Regular telephone follow-up after surgery lasted 3 years, showing that the patient remained in good condition without any relevant symptoms. By the time of writing of this manuscript, the patient had been undergoing surgical treatment for nearly 3 years without obvious clinical discomfort. In this patient, the tumor was large, and the profile was grayish yellow, solid, and soft. HE staining revealed an alternating zone of light and dark staining composed of many histiocytes, plasma cells and lymphocytes. Immunohistochemistry revealed that the tissue cells in the superficial staining area were rich in cytoplasm with scattered lymphocytes and characteristic deep movement. The tissue cells showed the remaining thymic epithelium and lymphocytes in the thymic tissue. Immunohistochemical results revealed CD68 ( +) S-100 ( +) CD1α (-) CD21 (-) PCK ( +) TDT ( +) (Fig. 2). Pathological examination revealed primary Rosai-Dorfman disease (RDD) in the thymus.

## Discussion

RDD, known as sinus histiocytosis with massive lymphadenopathy (SHML), is generally a rare benign disorder characterized by the proliferation of histiocytes [1]. First described in 1969, more than 600 cases have been reported worldwide. All age groups and both sexes are affected, but it is more common in children and adolescents and in males. The disease is characterized by painless, bilateral cervical lymphadenopathy in 90% of patients and may be accompanied by low-grade fever. Other common sites of nodal involvement include the axillary, inguinal, paraaortic, and mediastinal regions. Symptoms are also related to the presence and distribution of extranodal disease, which occurs in approximately 43% of patients with or without lymph node involvement. Common extranodal sites include the skin, central nervous system, bone, soft tissues, upper respiratory tract, and salivary glands [2]. There are very few reports of RDD in the thymus [2–5]. Osama [6] reported that most patients had multiple sites of involvement: of 10 patients, only one had pure lymphadenopathy, and all others had some combination of anatomic sites. All three patients with spinal involvement also had either intracranial involvement (*n* = 2) or head and neck involvement (*n* = 1). If Rosai-Dorfman disease is diagnosed at one site, other sites are also likely to be involved.

The underlying causes of RDD remain unclear. It has been considered a neoplastic, immune, or infectious process in various reports [7, 8]. Several viruses have been postulated to be associated with the onset of this disease. Epstein‒Barr virus and human herpes virus 6 have been detected by in situ hybridization in some patients with RDD [9]. Mehraein et al. [7] described four patients with RDD who had numerous parvovirus B19–positive cells detected in their tissues. B19 is associated with a variety of inflammatory vasculitides. Osama reported that one patient had concomitant RDD and Takayasu arteritis.

There are various reported RDD symptoms, including persistent fever, significant fatigue, poor appetite, noticeable weight loss, and a painless neck mass. However, some RDD patients without any symptoms have been diagnosed with unexpected findings of enlarged lymph nodes in the neck, mediastinum, or other sites, similar to the present case in our report.

The preoperative diagnosis of RDD is very difficult, especially when the lesion is located in the anterior mediastinum. It is difficult to distinguish RDD from conventional thymic tumors. There was no specificity in terms of tumor size, relationship with surrounding tissues or CT density. Preoperative biopsy of all thymic tumors is impractical and unnecessary.

However, in RDD, the thymic mass is composed of histiocytes, not thymic cells. In 1992, Kubota et al. [10] reported that macrophages (histiocytes) adjacent to a tumor show greater FDG uptake than viable tumor cells do. This finding supports our observation of intensely increased FDG uptake by a histiocytic proliferative lesion. Distinguishing RDD from malignant tumors may be difficult, even with FDG PET, because proliferative histiocytes in RDD have been observed to have higher FDG uptake than tumor cells. Osama A [6] reported that two of three patients with paranasal sinus involvement had aggressive disease with bony destruction. Such aggressive disease is difficult to differentiate from malignant conditions, making the diagnosis of RDD a mainly histologic, rather than radiologic, diagnosis.

The histopathology of RDD is very characteristic. Lymph nodes show massively distended sinuses, numerous large histiocytes with vesicular nuclei, distinct nucleoli and abundant pale cytoplasm and emperipolesis. Emperipolesis, a biological phenomenon defined as the active penetration of one cell by another that remains intact. Histiocytes are positive for the immunohistochemical stain S100 and some CD68 and are typically negative for CD1a. Laboratory findings in RDD patients include leukocytosis; neutrophilia; normocytic anemia; thrombocytosis; elevated CRP, ESR, and ferritin levels; and hypergammaglobulinemia [11]. However, these laboratory findings are unspecific.

RDD can easily be misdiagnosed because it mimics infections such as tubercular adenitis, reactive lymphadenopathies, and malignancies such as lymphoma and metastatic carcinoma, which all occur more frequently than RDD.

The course of the illness in patients with RDD is unclear. Classic symptoms, such as cervical lymphadenopathy, fever, and good general condition, may persist from several weeks to even a few years (the average duration is 3–9 months) [12]. Without treatment, relapsing–remitting RDD will occur in 70%, spontaneous regression in 20%, and progression of the disease in 10% of patients [13]. It is recommended that a clinical examination and laboratory tests be performed every 3–6 months during the first two years following diagnosis and then every year thereafter [13].

In 2011, the Histiocyte Society divided RDD patients into three major groups: 1) patients with sudden enlargement of the lymph nodes, in which spontaneous regression is observed, without any further recurrences – consistent with the best prognosis; 2) patients with immunological abnormalities, in which lymphadenopathy is more generalized – with the prognosis being worse; and 3) patients with extranodal site involvement and/or multinodal disease, with repeated relapses and remissions over a period of years – with the prognosis depending on the type and number of extranodal sites [14].

RDD in most cases is a benign disorder; however, multiorgan involvement or dysfunction and its association with immune dysfunction are poor prognostic indicators and may lead to death. The most common causes of reported deaths are immunological abnormalities, severe infections, surgical complications, complications after radiotherapy, and the compression of airways by enlarged lymph nodes [12, 15]. There are reports of RDD leading to lymphoma, amyloidosis, and consequent death from these diseases [15].

Because of the rarity of RDD and the limited experience in treating this disease, there are no commonly accepted therapeutic modalities. Because spontaneous remission can be observed in a considerable number of RDD patients, the “watch and wait” principle is recommended [13, 16] only if the diagnosis of this disease can be confirmed. Although the majority of patients can safely receive this “watch and wait” approach without any therapy, some patients whose vital organs, such as the airways or spinal cord, are moderately or severely compressed or obstructed require appropriate treatment [13, 14, 17].

Treatments for RDD include surgical resection, chemotherapy, radiotherapy, and steroids. Low-dose interferon is occasionally used in some patients [18]. Very few clinical trials on chemotherapy and radiotherapy for RDD have been carried out, and therefore, the effects of these methods are still ambiguous. According to the latest findings, radiotherapy has a better therapeutic effect than chemotherapy in RDD patients [14, 19]. Therefore, radiotherapy is considered the preferred procedure for patients with symptomatic RDD and for steroid-resistant patients [20, 21]. There are reports describing RDD patients with a high level of HHV-6/VZV antibody titers, in which there was a significant improvement after the application of acyclovir [14, 22, 23]. In other cases, partial remission was observed after thalidomide treatment [14, 19]. Surgery was also a reasonable option for RDD patients whose great vessels or airways were severely compressed; in particular, the diagnosis could not be ascertained before surgery. In the present report, RDD manifested as a solitary, unidentified lump in the thymic site, so we performed complete resection of the lesion. The patient has been in good condition for 3 years since the surgery and is still undergoing regular follow-up.

## Conclusions

RDD originating from the thymus is not distinctive in CT images, is very difficult to diagnose before surgery, and is easily misdiagnosed as a traditional mediastinal tumor. This is mainly because there is so little disease in this area that physicians are not aware of it. We have reported this case with the hope that clinicians will have a better understanding of this disease. We also hope to report more cases to facilitate the preoperative diagnosis and long-term evaluation of this rare tumor. According to our follow-up results, surgery is an effective means of treatment.

## Data Availability

No datasets were generated or analysed during the current study.
